# Aging and brain plasticity

**DOI:** 10.18632/aging.101514

**Published:** 2018-08-01

**Authors:** Lisa Pauwels, Sima Chalavi, Stephan P. Swinnen

**Affiliations:** 1 Movement Control and Neuroplasticity Research Group, Faculty of Movement and Rehabilitation Sciences, Group Biomedical Sciences, KU Leuven, Leuven, Belgium; 2 Leuven Brain Institute (LBI), KU Leuven, Leuven, Belgium

**Keywords:** aging, neuroplasticity, contextual Interference, Gamma-aminobutyric acid (GABA)

For a long time, it has been assumed that brain plasticity peaks at young age and then gradually decreases as one gets older. This is also underscored by the expression that one cannot teach an old dog new tricks, implying that people who have become used to doing things in a particular way will not easily abandon their habits and change their behavior. Interestingly, thanks to tremendous advances in medical imaging techniques for assessment of brain structure and function, mounting evidence for lifelong brain plasticity has been generated over the past years.

Practice leads to improvement in and refinement of performance on motor - or any other -tasks and this dynamic behavioral process is associated with altered brain activity, occurring in a similar manner in young and older adults [1]. Besides functional brain changes, practice also induces structural changes, such as alterations in regional brain grey and white matter structures that are typically recruited during task performance [2].

In the context of practice-induced task learning, a key question is how brain plasticity can be optimized and this is an even more important consideration for older adults. Noninvasive brain stimulation techniques have been utilized to induce and optimize mechanisms of neuroplasticity during rest or during task practice via alteration of cortical excitability processes [3]. However, the effectiveness of such approaches has been questioned lately and we would argue that the gold standard to elicit brain plasticity is to practice new tasks intensively and to organize the training epochs in such a way that skill learning and retention are maximized. A critical requirement for neuroplasticity to emerge is to make the practice context sufficiently difficult for the learner. One way to challenge the environmental context is to confront learners with practicing more than one task within each practice session. More specifically, rather than performing subtasks in a sequential or blocked manner, one after the other (less challenging), one can also apply a more demanding random practice regime such that learners have to switch tasks from trial to trial during practice (more challenging). The latter condition has led to the apparent paradox that reduced performance levels are obtained during the training phase but better long-term retention and memory formation of the skill are observed at later stages as a result of more profound inter-task information processing strategies. This is generally known as ‘contextual interference’ (CI). Even though CI seemingly induces complication of the learning environment, it has been shown that older adults can equally cope with this increased contextual complexity as young adults do and that it benefits longer-term skill retention [4,5].

Using magnetic resonance spectroscopy (MRS), we explored the neurochemical basis of the CI effect via determination of the practice-induced modulation of gamma-aminobutyric acid (GABA), i.e. the chief inhibitory neurotransmitter that also plays a major role in brain plasticity [6]. Young and older participants were trained on 3 variations of a bimanual visuomotor tracking task over 3 days, according to either a blocked or random practice schedule. A retention test was carried out 6 days later. With MRS, we determined the GABA levels before and after training in primary sensorimotor cortex and occipital cortex, because our previous work showed considerable brain activity in both visual and motor processing regions, in agreement with the unique visuomotor signature of this tracking task [7]. We found that (1) the behavioral data confirmed the typical contextual interference effects, i.e., random as compared to blocked practice led to temporary inferior performance levels during the acquisition phase but superior skill retention in both age groups, (2) the MRS data demonstrated a training-induced decrease in occipital GABA level during random practice but an increased GABA level during blocked practice and this effect was even more pronounced in older adults [6]. These findings are critical for a better understanding of neuroplasticity in older adults. First, the data suggest that older adults can indeed cope with more complex random practice contexts (a form of contextual enrichment) that challenge their instantaneous performance but boost their learning potential and skill retention. Second, training-induced modulations in GABA appear to be a function of degree of contextual challenge and this effect is even amplified by aging. This modulatory capability is preserved in spite of the fact that initial GABA levels were lower in older as compared to young adults [6]. This is important because it is reflective of training-induced neuroplasticity in older adults. More specifically, a decrease in GABA levels is indicative of a release from inhibition to promote mechanisms of long-term potentiation, cortical plasticity and learning [6,8]. Whereas previous work reported this GABA modulation to occur in the motor cortex [Floyer-Lea A, et al. 2006] we observed it in the broader occipital territory, containing brain areas involved in processing of the stimulus structure and the movement-generated visual feedback, made available during practice (such as primary and secondary visual cortex and posterior precuneus), as exemplified by task-related functional magnetic resonance imaging [7]. Conversely, the less demanding blocked-practice schedule consisted of repeated presentation of stimuli, resulting in attenuated neuronal responses (repetition suppression) and enhanced GABA levels.

These data provide additional confirmation for task-training induced lifelong plasticity. New motor and other skills can be acquired at any age even though the progress may be somewhat attenuated in older as compared to young populations. The importance of these results should not be underestimated. We live in a highly dynamic society in which dramatic technical developments are implemented at a rapid pace. This forces citizens to abandon their pre-existing/old habits and replace these by new ones, continuously challenging the adaptability and flexibility of their brains. In view of the demographic evolution of society, characterized by a steadily increasing proportion of older adults, the evidenced lifelong brain plasticity provides a critical foundation for a sustained role of older adults in society and for securing prolonged functional independence and quality of life. Society needs to provide the right context in which older adults remain challenged and encouraged to adapt to new contexts such that the negative consequences of age-related brain degeneration are reduced or even reversed and healthy brains are promoted.
